# THE EFFECTS OF SIMULATION-BASED EMPATHY ENHANCEMENT PROGRAM FOR CARER OF THE ELDERLY ON COUNSELLING

**DOI:** 10.1093/geroni/igz038.3354

**Published:** 2019-11-08

**Authors:** Sangmi Park, Tae Hui Kim, Tae Rim Um, Kyuwon Lee, Jisoo Jung, Myounghee Hong, Soyeon Choi, Areum Han

**Affiliations:** 1 Wonju Severance Christian Hospital, Wonju, Korea, Republic of; 2 Wonju Severance Christian Hospital, Wonju, Gangwon, Korea, Republic of; 3 Yonsei University, Wonju, Gangwon, Korea, Republic of

## Abstract

Empathy of the caregiver can influence both the caregiver’s performance and the receiver’s enhanced life. The aim of this study is to examine whether Simulation-based Empathy Enhancement program for the Carer of the Elderly (SEE-C) is effective in increasing care receivers’ session satisfaction and positive emotional change. We developed SEE-C by modifying the Dementia Live(TM) program and adding with a brief mindfulness. The effect on counselling was assessed using the Session Evaluation Questionnaire (SEQ), which is self-report tool asking the client about their experience with the session just ended. A total of 100 older adults living alone were interviewed by caregivers who experienced SEE-C (n=12) and by non-experienced (n=12). Participants in this study were randomly assigned to each of the two caregiver groups, and were interviewed about demographics, health and emotional status, and lifestyle using the same protocols. Analysis of covariance was conducted, controlling variables of age of subjects and caregivers’ months of career, which were found to differ significantly between the two groups. Among the four subcategories of SEQ, the experimental group reported significantly higher scores than the control group in three subcategories of session-depth (F(1, 96)=9.647, P=.002), session-smoothness (F(1, 96)=13.699, p<.001), emotion-positive (F(1, 96)=18.056, p<.001), with the exception of emotion-alertness (F(1, 96)=0.366, p=.546). These results suggest that SEE-C could have a positive impact on interviewing the elderly in terms of improving the capacity of the interviewer and raising the satisfaction of the interviewee.

